# Incidence of major cardiovascular events in patients with metabolic dysfunction‐associated steatotic liver disease in the general population

**DOI:** 10.1002/ejhf.70053

**Published:** 2025-09-26

**Authors:** Yvonne Huber, Lea Hofmann, Jürgen H. Prochaska, Thomas Koeck, Julian Chalabi, Norbert Pfeiffer, Manfred Beutel, Konstantin Strauch, Karl J. Lackner, Oliver Tüscher, Thomas Münzel, Matthias M. Weber, Julia Weinmann‐Menke, Philipp Wild, Peter R. Galle, Jörn M. Schattenberg

**Affiliations:** ^1^ Department of Medicine I University Medical Center of the Johannes Gutenberg University Mainz Mainz Germany; ^2^ Metabolic Liver Research Program, Department of Medicine I University Medical Center of the Johannes Gutenberg University Mainz Mainz Germany; ^3^ Preventive Cardiology and Preventive Medicine, Department of Cardiology University Medical Center of the Johannes Gutenberg University Mainz Mainz Germany; ^4^ German Center for Cardiovascular Research, partner site Rhine Main Mainz Germany; ^5^ Center for Thrombosis and Hemostasis, University Medical Center of the Johannes Gutenberg University Mainz Mainz Germany; ^6^ Department of Ophthalmology University Medical Center of the Johannes Gutenberg University Mainz Mainz Germany; ^7^ Department of Psychosomatic Medicine and Psychotherapy University Medical Center of the Johannes Gutenberg University Mainz Mainz Germany; ^8^ Institute of Medical Biostatistics, Epidemiology and Informatics, University Medical Center of the Johannes Gutenberg University Mainz Mainz Germany; ^9^ Institute of Clinical Chemistry and Laboratory Medicine, University Medical Center of the Johannes Gutenberg University Mainz Mainz Germany; ^10^ Institute of Molecular Biology Mainz Mainz Germany; ^11^ Department of Internal Medicine II Saarland University Medical Center, Homburg and Saarland University Saarbrücken Germany; ^12^ PharmaScienceHub (PSH) Saarbrücken Germany

**Keywords:** Major cardiovascular events, Cardiovascular diseases, Population‐based study, Fatty liver disease, Metabolic dysfunction‐associated steatotic liver disease

## Abstract

**Aims:**

Major adverse cardiovascular events (MACE) related to cardiovascular disease are a major cause of death in patients with metabolic dysfunction‐associated steatotic liver disease (MASLD). We explored the impact of MASLD on incident MACE and overall mortality in the general population in Germany.

**Methods and results:**

A total of 14 575 patients were included for the analysis. Elevated liver enzymes were present in 21.7% and MASLD, defined with a positive fatty liver index (FLI) in the absence of relevant alcohol use, was detected in 37% of participants. MACE were defined as a three‐item composite endpoint of acute myocardial infarction (AMI), stroke and cardiovascular mortality (3‐point MACE) and extended MACE (eMACE) including MACE criteria, incident atrial fibrillation and pulmonary embolism. In the group with a positive FLI (≥60) a higher rate of male sex and a higher age as well as a higher prevalence of metabolic and cardiovascular risk factors compared to the group with a negative FLI (<30) were present. At 5 years of follow‐up, 475 patients (3.7%) developed 3‐point MACE and 577 (4.9%) developed eMACE. In the subgroup with MASLD, the incidence of eMACE was higher (7.1% vs. 3.7%; *p* < 0.0001). Using Cox regression analysis with a stepwise adjustment strategy, we were able to show an independent prediction of MACE and eMACE by hepatic steatosis under consideration of various confounders. The presence of MASLD was associated with an increased risk of developing MACE by 62.3% (*p* < 0.0001) and eMACE by 44.0% (*p* < 0.0001). Importantly, MASLD was associated with an increased risk for all‐cause mortality (hazard ratio 1.55; *p* < 0.0001).

**Conclusions:**

Metabolic dysfunction‐associated steatotic liver disease is an independent risk factor for MACE and is associated with a significantly increased risk of all‐cause mortality. In the management of patients with cardiovascular risk, identification of MASLD can potentially refine their disease trajectory.

## Introduction

Metabolic dysfunction‐associated steatotic liver disease (MASLD) is the most common chronic liver disease today, with an estimated prevalence of 30% among the global adult population.^1^, ^2^ In recent decades, the number of patients has steadily increased, accompanied by the growing epidemic of obesity and type 2 diabetes mellitus in the world.^3^, ^4^ According to the Global Burden of Disease study, MASLD is one of the leading contributors to the worldwide burden of disease and disability caused by chronic liver disease.^5^ The related socio‐economic costs in Western countries are immense^6^, ^7^ – recent data from the United States' United Network of Organ Sharing (UNOS) suggest that MASLD will soon become the leading indication for liver transplantation in the United States.^8^, ^9^, ^10^ To emphasize the metabolic dysfunction underlying the pathogenesis of the disease, the former term of non‐alcoholic fatty liver disease (NAFLD) has been replaced by a new fatty liver disease nomenclature proposed by an international Delphi consensus statement.^11^ MASLD is now defined by the presence of hepatic steatosis in patients with at least one out of five cardiometabolic risk factors and no other likely cause of steatotic liver disease like excessive alcohol consumption or drug‐induced liver injury.^12^ The five risk factors include overweight or obesity, dysglycaemia or type 2 diabetes, hypertension, elevated plasma triglycerides, and reduced high‐density lipoprotein (HDL) cholesterol.^12^, ^13^ MASLD must be differentiated from alcohol‐associated liver disease (ALD) and an overlap syndrome called MetALD, which represent new subcategories under the overarching term of steatotic liver disease (SLD).^14^, ^15^ MASLD encompasses the entities of metabolic dysfunction‐associated steatotic liver (MASL) and metabolic dysfunction‐associated steatohepatitis (MASH), which replace the former terms of NAFL and NASH.^14^, ^16^ Retrospective studies show high concordance rates of ≥99% between the MASLD and NAFLD patient cohorts.^17^ MASL is characterized by macro vesicular steatosis in ≥5% of hepatocytes, whereas MASH additionally shows aspects of lobular inflammation and cellular injury.^18^ MASH is often accompanied by hepatic fibrosis and can eventually progress into liver cirrhosis with its various complications like acute‐on‐chronic liver failure or hepatocellular carcinoma.^19^ The most common cause of death in MASLD patients is cardiovascular disease (CVD) followed by extrahepatic malignancy.^20^, ^21^, ^22^ CVD include a wide spectrum of pathological conditions affecting the heart and the blood circulatory system.^23^ They can result in acute, potentially life‐threatening events like acute myocardial infarction or ischaemic stroke, which are summarized by the umbrella term of major adverse cardiovascular events (MACE).^24^ However, they can also lead to chronic organ dysfunction like congestive heart failure or vascular dementia, which contribute to reduced quality of life, disability and premature death.^25^ A central pathogenic mechanism is the atherosclerotic degeneration of small and large blood vessels (atherosclerotic CVD) and a lot of risk factors are well‐known including dyslipidaemia, diabetes, hypertension and smoking.^26^ In the past years, observational studies in different countries have been published, which showed a significant association between MASLD as pre‐existing condition and the development of CVD.^22^, ^27^, ^28^, ^29^ In 2021, a systematic meta‐analysis of 36 studies involving more than 5.8 million patients has identified MASLD as an independent risk factor for fatal and non‐fatal CVD. Also, the risk increased with the severity of MASLD and especially the degree of fibrosis.^30^ The current analysis was performed in order to get further insight into epidemiological characteristics of MASLD in the German general population as well as to evaluate the impact of MASLD on incident MACE over 5‐year follow‐up.

## Methods

### Patients and study description

We analysed cross‐sectional data of 15 010 participants enrolled in the Gutenberg Health Study (GHS) between April 2007 and April 2012 and whose data were available at the time of follow‐up after 5 years. The GHS is a single‐centre, population‐based, prospective, and observational cohort study in the Rhine‐Main Region in Western Germany. Its design has been described in detail.^31^ Individuals between the ages of 35 and 74 years were selected randomly from local governmental registries with a sampling procedure that was stratified for sex, residential area (urban vs. rural), and decades of age. All invited participants had the opportunity to decline participation and provide a reason for doing so. The GHS has obtained ethical approval from the local ethics committee and has been approved by both the local and federal data safety commissioners.

### Baseline and 5‐year follow‐up examination

At the baseline examination and at the 5‐year follow‐up examination in the study centre, clinical variables and prevalent classical comorbidities were evaluated, along with a computer‐assisted personal interview, laboratory examinations using venous blood samples, and blood pressure and anthropometric measurements. All examinations were conducted according to standard operating procedures by certified medical technical assistants.

#### Anthropometric data

Weight, height, waist circumference (WC), and hip circumference were measured following a standardized manual. To measure WC, a tape measure was placed midway between the lowest rib and the pelvis during expiration. A WC of <94 cm in men and <80 cm in women was considered within the normal range as recommended by the International Diabetes Federation.^32^

#### Presence of comorbidities

Body mass index (BMI) was calculated as weight (kg)/height (m)^2^. Obesity was defined as BMI ≥30 kg/m^2^, while overweight was defined as BMI >25–<30 kg/m^2^. Type 2 diabetes was defined based on self‐reported physician diagnosis, medication use, fasting plasma glucose ≥126 mg/dl after an overnight fasting of at least 8 h or rather a blood glucose level of ≥200 mg/dl after a fasting period <8 h. Glycated haemoglobin (HbA1c) values were categorized into three groups: low (HbA1c <5.7%), intermediate (HbA1c 5.7–6.5%), and high (HbA1c >6.5%). HbA1c values >6.5% were considered elevated. Hypertension was defined based on self‐reported physician diagnosis, medication use, systolic blood pressure ≥140 mmHg, or diastolic blood pressure ≥90 mmHg in the second and third standardized measurement after 8 and 11 min of rest. Dyslipidaemia was defined based on self‐reported physician diagnosis, medication use, or a low‐density lipoprotein (LDL)/HDL ratio of >3.5. The presence of the metabolic syndrome was defined according to the criteria established in the joint scientific statement for harmonizing the metabolic syndrome.^33^ Pre‐existing chronic liver disease was self‐assessed by replying to the following question: ‘Have you ever been medically diagnosed with liver disease?’ The study did not specifically test for chronic viral hepatitis or autoimmune liver disease. As the reported prevalence of hepatitis C or B virus in Germany is 0.3% at the population level, this will not significantly alter the findings and conclusions. Smoking was categorized into non‐smokers (no nicotine and ex‐nicotine consumption) and smokers (including occasional consumption of less than one cigarette per day). Alcohol consumption was recorded using a questionnaire. This included quantitating regular alcohol use and the types of drinks in detail with breakdown on weekdays and weekend. The specified quantities were then converted to alcohol in g/week. Relevant alcohol consumption was defined as an average daily intake of ≥20 g of pure alcohol for women and ≥30 g for men.^11^ High regular daily consumption was defined as an intake of ≥40 g for women and ≥60 g for men leading to exclusion from the study analysis, which was aligned with MASLD diagnostic criteria.^34^

#### Reference values for elevated liver enzymes

References values were as follows: gamma‐glutamyl transferase (gGT) >64 U/L in men and >36 U/L in women, alanine aminotransferase (ALT) >50 U/L in men and >35 U/L in women, aspartate aminotransferase (AST) >35 U/L in men and >31 U/L in women. One surrogate marker for hepatic steatosis (fatty liver index [FLI])^35^ and one for fibrosis (fibrosis‐4 [FIB‐4] score; lower‐cut off 1.3; upper‐cut off 2.67) were evaluated, and advanced fibrosis was defined based on previously published cut‐offs.^36^, ^37^ FLI is calculated as: FLI = (e^0.953×loge (triglycerides)+0.139×BMI+0.718×loge(ggt)+0.053×WC −15.745^)/(1 + e ^0.953×loge (triglycerides)+0.139×BMI+0.718×loge(ggt)+0.053×WC −15.745^) × 100.^35^ FLI was seen as positive with values ≥60. Changes in FIB‐4 categories were examined between baseline and the 5‐year follow‐up. In epidemiological studies, FLI has been used repeatedly and is a validated approach.^38^, ^39^ The presence of MASLD was defined as the presence of steatosis of the liver with a positive FLI and the exclusion of other chronic liver disease or high alcohol consumption.^11^

#### Major adverse cardiovascular events

Major adverse cardiovascular events were defined as a three‐item composite endpoint of acute myocardial infarction, stroke and cardiovascular mortality as originally proposed by the United States Food and Drug Administration (FDA).^24^, ^40^ Extended MACE (eMACE) included primary MACE plus incident atrial fibrillation and pulmonary embolism.^41^, ^42^ A preexisting MACE or eMACE at baseline was an exclusion criterion for corresponding statistical analyses.

### Detailed study information

**Figure 1:**
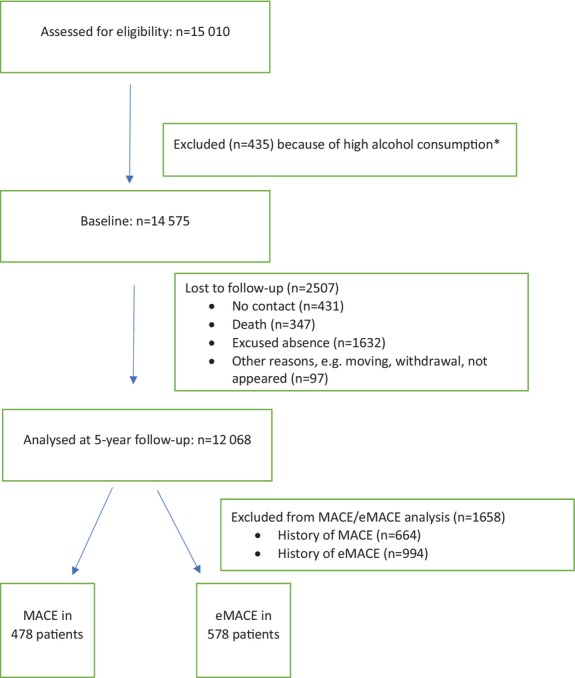
Participant flow chart. eMACE, extended major adverse cardiovascular events; MACE, major adverse cardiovascular events. *High alcohol consumption: defined as a daily intake of ≥40 g for women and ≥60 g for men.

Of the 15 010 participants in the GHS baseline cohort, 435 patients had to be excluded from the analyses due to excessive alcohol consumption. Within 5 years of follow‐up (median follow‐up time: 5.03 years), 2507 participants were lost to follow‐up, thus leaving 12 068 participants for analysis (*Figure* *1*). The endpoints MACE and eMACE were analysed using a time‐to‐event approach. Medical records, as well as information from physicians and from the study participants themselves, were collected and submitted to a team of experts. Mortality data were obtained through quarterly queries to the official registry offices and the mortality registry of Rhineland‐Palatine, Germany, that cover all deaths. Models for death and mortality were available for 15 years of observation.

The team of experts was composed of two physicians and an epidemiologist and evaluated the documents in regular meetings. The team reviewed the information to identify diseases relevant to the study.^31^

Baseline characterization of the study cohort was also standardized according to European Standard Population 1976.^43^

### Statistical analysis

Statistical analysis was conducted using R version 4.1.0 (R Foundation for Statistical Computing, Vienna, Austria; https://www.R‐project.org/). Continuous variables are reported as mean values (standard deviation) or as median (interquartile range) for skewed distributions. Categorical variables were described as absolute and relative frequencies. Adjusted proportional hazards models with consideration of competing events were used to analyse the relationship between the presence of hepatic steatosis and time‐to‐event outcome. All *p*‐values correspond to two‐tailed tests and the threshold of significance was set to 0.05. No adjustments were made for multiple comparisons, as this was an exploratory study. Consequently, *p*‐values should be interpreted with caution and in conjunction with effect size estimates.

## Results

### Patient characteristics

A total of 15 010 participants were enrolled in the GHS and after exclusion of patients with preexisting MACE or harmful alcohol consumption, 14 575 patients were final analysed. The mean age in this cohort was 54.9 ± 11.1 years with equal gender distribution. Baseline characteristic including demographic data, laboratory values and prevalence of comorbidities are presented in *Table* *1*. Metabolic and cardiovascular risk factors were significantly more frequent in men, with arterial hypertension in 54.0% of men and 44.6% of women (*p* < 0.0001). Likewise, diabetes (11.3% vs. 7.3%; *p* < 0.0001), obesity (26.2% vs. 24.2%; *p* < 0.01), dyslipidaemia (43.2% vs. 26.0%; *p* < 0.0001), hyperuricemia (12.9% vs. 6.1%; *p* < 0.0001), and metabolic syndrome (29.0% vs. 17.0%; *p* < 0.0001) were more prevalent in men. A history of liver disease was more prevalent in women (9.0% vs. 7.6%; *p* < 0.01) as well as cancer (10.1% vs. 8.0%; *p* < 0.0001). Baseline characterization of the study cohort was also standardized according to European Standard Population 1976 (online supplementary *Table* *S1*), where comparable results were shown with increased metabolic and cardiovascular risk factors in men.

**Table 1:** Baseline characterization of the study cohort and fatty liver index according to demographic characteristics and comorbidities

Parameter	All^a^ (*n* = 14 575)	FLI <60 (*n* = 9160)	FLI ≥60 (*n* = 5373)	*p*‐value (FLI <60 vs. FLI ≥60)
Age (years)	54.9 ± 11.1	53.5 ± 11.2	57.4 ± 10.6	< 0.0001
Female sex	7287 (50)	5421 (59.2)	1841 (34.3)	< 0.0001
BMI (kg/m^2^)	26.6 (23.9–30.0)	24.7 (22.7–26.6)	31.1 (28.8–34.0)	< 0.0001
Waist circumference (cm)	94.4 ± 13.9	86.7 ± 9.1	107.6 ± 10.2	< 0.0001
Obesity	3670 (25.2)	325 (3.5)	3332 (62.0)	< 0.0001
Diabetes mellitus type 2	1348 (9.3)	379 (4.1)	963 (17.9)	< 0.0001
Hypertension	7183 (49.3)	3417 (37.3)	3741 (69.7)	< 0.0001
Dyslipidaemia	5030 (34.6)	2294 (25.1)	2725 (50.8)	< 0.0001
Hyperuricemia	1340 (9.5)	432 (4.1)	903 (17.5)	< 0.0001
Metabolic syndrome	3347 (23.0)	725 (7.9)	2619 (48.7)	< 0.0001
Chronic liver disease	1196 (8.3)	612 (6.7)	580 (10.9)	< 0.0001
Cancer	1311 (9.0)	801 (8.8)	510 (9.5)	0.13
Smoker	2786 (19.2)	1781 (19.5)	998 (18.6)	0.2
Triglycerides (mg/dl)	105 (78–147)	88 (68–114)	147 (112–196)	<0.0001
Cholesterol (mmol/L)	221 ± 41	219 ± 40	223 ± 42	<0.0001
HDL (mg/dl)	57 ± 16	62 ± 16	49 ± 12	<0.0001
LDL (mg/dl)	139 ± 36	138 ± 35	141 ± 36	<0.0001
ALT (U/L)	32 (26–42)	29 (25–36)	40 (31–51)	<0.0001
AST (U/L)	25 (22–29)	24 (21–28)	27 (23–32)	<0.0001
gGT (U/L)	24 (17–37)	19	36	< 0.0001

Continuous variables are shown as mean ± standard deviation and tested with *t*‐test, or as median (interquartile range) and tested with U test. Binary variables are described through relative and absolute frequencies and tested with chi‐squared test.

ALT, alanine aminotransferase; AST, aspartate aminotransferase; BMI, body mass index; FLI, fatty liver index; gGT, gamma‐glutamyl transferase; HDL, high‐density lipoprotein; LDL, low‐density lipoprotein.

^a^ The FLI data available for 14 533 patients.

### Prevalence of elevated liver enzymes and surrogate scores of hepatic steatosis

Elevated liver enzymes at baseline were detected in one out of five participants with increased ALT levels in 21.7%, increased AST levels in 12.4% and elevated gGT levels in 13.4%. Considering sex specific cut‐offs, ALT > upper limit of normal (ULN) was seen in 20.9% of men and 22.4% of women (*p* < 0.05). AST was elevated > ULN in 13.8% of men and 11.0% of women (*p* < 0.0001) and gGT > ULN in 12.3% of men and 14.4% of women (*p* < 0.001).

The FLI as surrogate score of hepatic steatosis was positive (FLI ≥60) in 37% (*n* = 5373) of the participants at baseline. In the subgroup with a positive FLI, men were overrepresented compared to women (65.7% vs. 34.3%; *p* < 0.0001). Regarding age, FLI was more frequently positive in elderly persons (≥55 vs. <55 years: 43.8% vs. 29.6%; *p* < 0.0001) at baseline. Also, participants with positive FLI showed significantly more metabolic and cardiovascular risk factors than participants with negative FLI (*Table* *1*). In the standardized study cohort, there was a prevalence of hepatic steatosis (FLI ≥60) of 34.6% (*n* = 5045).

Fibrosis‐4 was used as non‐invasive surrogate score for hepatic fibrosis. At baseline 0.8% (*n* = 116) of patients exhibited a FIB‐4 above the upper cut‐off of 2.67, and increasing FIB‐4 was significantly more frequent in men (*p* < 0.001).

### Major adverse cardiovascular events

At 5‐year follow‐up, 478 patients (3.7%) developed a MACE. Patients with a positive FLI at baseline showed significantly more MACE than those with a negative FLI (5.7% vs. 2.6%; *p* < 0.0001). An eMACE was even more frequent in both groups: in total 578 patients (4.9%) developed eMACE with more eMACE in the group with a positive FLI at baseline (7.1% vs. 3.7%; *p* < 0.0001). We applied a proportional hazards model accounting for mortality as a competing event, hence being able to further specify whether people who have been censored during the time to event are further at risk or not, which enhances statistical interpretability. With regard to eMACE there was only one event and for MACE there were 164 competing events. Adjusting for age and sex, the presence of hepatic steatosis was associated with an increased risk of developing MACE by 62.3% (hazard ratio [HR] 1.623, 95% confidence interval [CI] 1.31–2.01; *p* < 0.0001) and eMACE by 44.0% (HR 1.440, 95% CI 1.21–1.72; *p* < 0.0001). Furthermore, hepatic steatosis was also associated with an increased risk for all‐cause mortality (HR 1.55, 95% CI 1.41–1.71; *p* < 0.0001) in the follow‐up time of 15 years.

A Cox regression model with a stepwise adjustment strategy was developed to identify the risk factors mediating MACE and eMACE. First, we conducted a basic model with adjustments for sex and age. In a next step, we additional adjusted for further risk factors like waist‐to‐height ratio, arterial hypertension and other risk factors (*Table* *2*). In the analysis, a significant association of hepatic steatosis with MACE and eMACE was found, even after adjusting for single, known risk factors. The increase in HR, when adjusting for factors like arterial hypertension, dyslipidaemia and diabetes mellitus in *Table* *2* implies that hepatic steatosis, if present without these factors, represents a higher risk factor for MACE. In a Cox regression model adjusted simultaneously for all relevant variables for MACE and eMACE, we see that FLI is no longer significantly associated with MACE and eMACE (*Table* *2*). This indicates that the factors remaining relevant in the model explain the association between hepatic steatosis and MACE. Interestingly, in this model, the risk factors BMI for MACE and ‘diabetes mellitus for eMACE also lose their significance in predicting the outcome.

**Table 2:** Cox regressions stepwise analysing risk factors for major adverse cardiovascular events (MACE) and extended MACE

	HR (95% CI), *p*‐value	
	MACE	eMACE
**Hepatic steatosis (FLI ≥ 60)**		
Basic model: adjusted for sex and age	1.62 (1.31–2.01), ***p* < 0.0001**	1.44 (1.21–1.72), ***p* < 0.0001**
Additionally adjusted for waist‐to‐height ratio	1.33 (1.01–1.76), ***p* = 0.046**	1.098 (0.87–1.39); *p* = 0.44
Additionally adjusted for arterial hypertension	1.46 (1.18–1.81), ***p* < 0.001**	1.31 (1.10–1.57), ***p* < 0.01**
Additionally adjusted for dyslipidaemia	1.52 (1.23–1.88), ***p* < 0.0001**	1.38 (1.16–1.65); ***p* < 0.001**
Additionally adjusted for diabetes mellitus	1.53 (1.23–1.89), ***p* < 0.001**	1.41 (1.18–1.69); ***p* < 0.001**
**Adjusted for all relevant variables for MACE and eMACE**		
Hepatic steatosis (FLI ≥60)	1.16 (0.87–1.54), *p* = 0.32	0.97 (0.76–1.23), *p* = 0.78
Sex	0.50 (0.40–0.63), ***p* < 0.0001**	0.56 (0.47–0.67), ***p* < 0.0001**
Age	1.07 (1.06–1.08), ***p* < 0.0001**	1.07 (1.06–1.08), ***p* < 0.0001**
BMI	1.04 (0.99–1.10), *p* = 0.09	1.07 (1.03–1.11), ***p* < 0.01**
Waist‐to‐height ratio	0.26 (0.01–7.47), *p* = 0.43	0.20 (0.01–3.36), *p* = 0.26
Arterial hypertension	1.57 (1.22–2.03), ***p* < 0.001**	1.44 (1.17–1.76), ***p* < 0.001**
Dyslipidaemia	1.45 (1.18–1.79), ***p* < 0.001**	1.29 (1.09–1.54), ***p* < 0.01**
Diabetes mellitus	1.38 (1.05–1.80), ***p* = 0.019**	1.03 (0.80–1.33), *p* = 0.82

BMI, body mass index; CI, confidence interval; FLI, fatty liver index; HR, hazard ratio.

**Figure 2:**
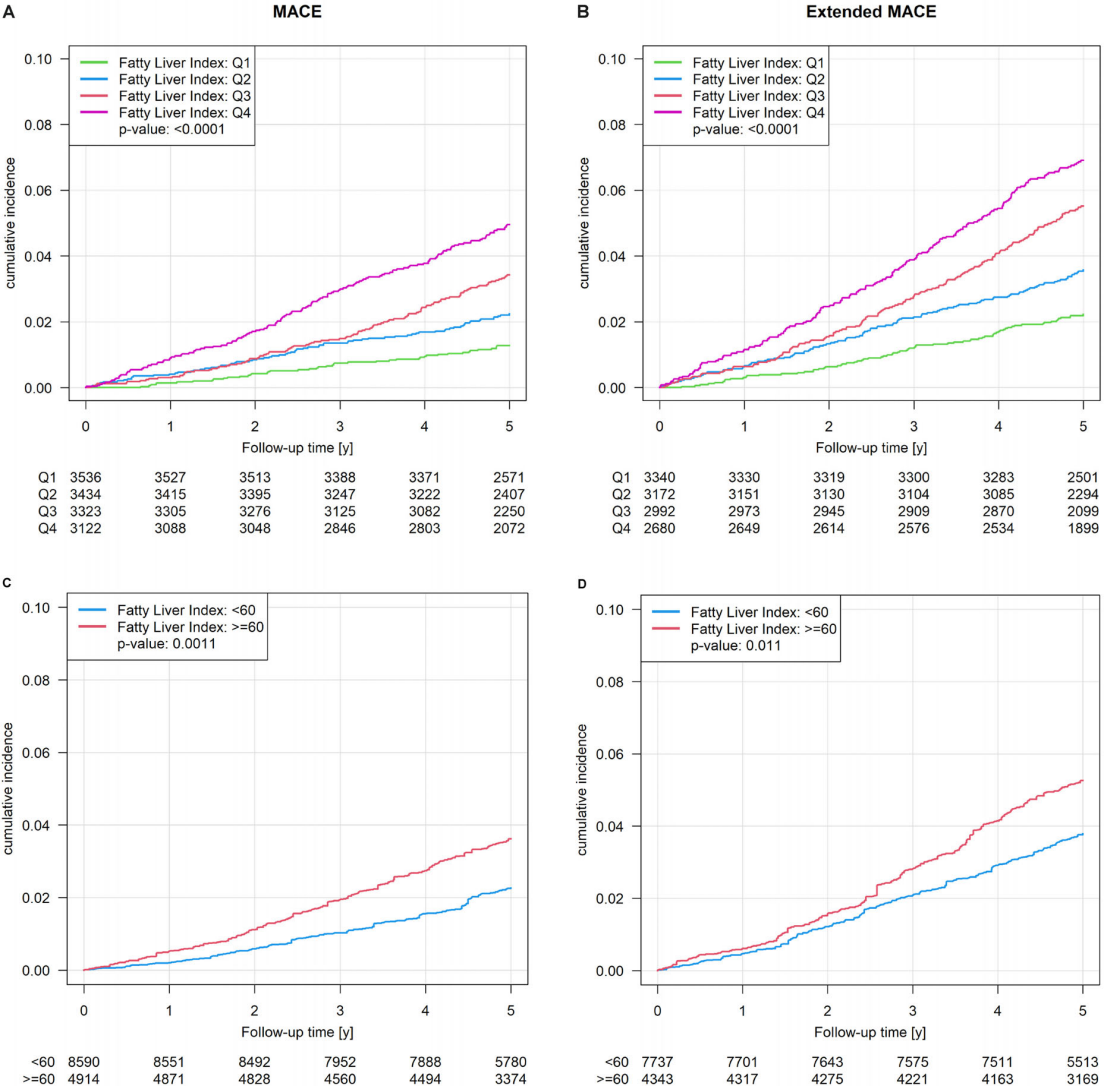
(*A*, *B*) Cumulative incidence of developing major adverse cardiovascular events (MACE) and extended MACE associated with the presence of hepatic steatosis. (*C*, *D*) Cumulative incidence of developing MACE and extended MACE associated with the presence of hepatic steatosis adjusted for waist‐to‐height ratio, arterial hypertension, dyslipidaemia and type 2 diabetes mellitus. Quartiles: Q1 = <18.23; Q2 = 18.23–<44.11; Q3 = 44.11–<74.72; Q4 = ≥74.72. *P*‐value: log‐rank test.

**Figure 3:**
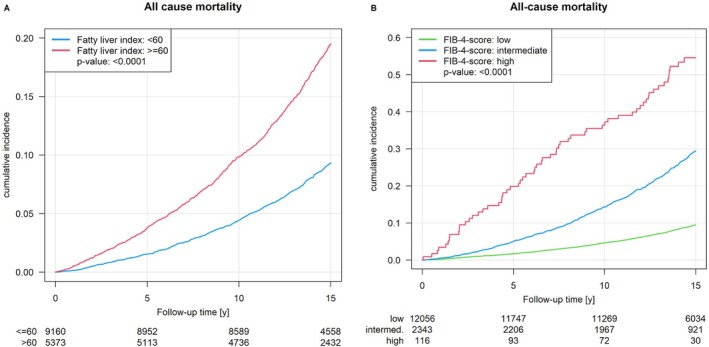
Cumulative incidence of all‐cause mortality associated with the presence of hepatic steatosis (*A*) and hepatic fibrosis (*B*). *P*‐value: log‐rank test.

The presence of hepatic steatosis showed an increased cumulative incidence of developing MACE (*Figure* *2A*) and eMACE (*Figure* *2B*), even after considering waist‐to‐height ratio, arterial hypertension, dyslipidaemia and type 2 diabetes mellitus as confounders (*Figure* *2C,D*) and an increased cumulative incidence of all‐cause mortality for 15 years of observation (*Figure* *3A*).

To examine whether the association between MASLD and MACE differs between obese and non‐obese individuals, we performed a stratified analysis using a basic model with sex and age and an interaction term for the effect of FLI on outcome in obese (BMI ≥30 kg/m^2^) versus non‐obese (BMI ≤30 kg/m^2^) individuals. The analysis revealed a significant interaction for both outcomes, MACE and eMACE. The effect of FLI on outcome was similar in both models with an approximately 1.02 times larger effect in obese than in non‐obese individuals (*Table* *3*). With regard to hepatic fibrosis, there was an increased risk of developing eMACE in patients with high FIB‐4 score compared to those with low FIB‐4 score (HR 2.15, 95% CI 1.11–4.18; *p* = 0.02), but no increased risk of developing MACE (HR 1.87, 95% CI 0.94–3.73; *p* = 0.07). An increased risk of all‐cause mortality in patients with a high FIB‐4 score (HR 2.53, 95% CI 1.94–3.31; *p* < 0.0001) and an indeterminate FIB‐4 (HR 1.25, 95% CI 1.12–1.39; *p* < 0.0001) compared the low FIB‐4 category was observed. An increased cumulative incidence of all‐cause mortality in the presence of hepatic fibrosis was observed (*Figure* *3B*).

**Table 3:** Impact of fatty liver index on major adverse cardiovascular events (MACE) and extended eMACE in regard to body mass index

	BMI <30 kg/m^2^	BMI ≥30 kg/m^2^
MACE		
Sex (women)	0.51 (0.38–0.68), *p* < 0.0001	0.58 (0.40–0.84), *p* < 0.01
Age	2.22 (1.94–2.55), *p* < 0.0001	1.98 (1.63–2.40), *p* < 0.0001
FLI	1.01 (1.00–1.01), *p* = 0.02	1.03 (1.02–1.05), *p* < 0.001
Interaction term of FLI in obese vs. non‐obese	1.023 (1.01–1.04), *p* = 0.008	
eMACE		
Sex (women)	0.59 (0.46–0.75), *p* < 0.0001	0.65 (0.47–0.88), *p* < 0.01
Age	2.15 (1.93–2.41), *p* < 0.0001	1.76 (1.51–2.06), *p* < 0.0001
FLI	1.00 (0.99–1.01), *p* = 0.08	1.03 (1.01–1.04), *p* < 0.001
Interaction term of FLI in obese vs. non‐obese	1.02 (1.01–1.03), *p* = 0.004	

Data presented as hazard ratio (95% confidence interval).

BMI, body mass index; FLI, fatty liver index.

To assess the clinical utility of MASLD in cardiovascular risk stratification, we evaluated whether adding MASLD to established risk prediction models improves predictive performance. Therefore, a comparison between a baseline model with Systematic COronary Risk Evaluation 2 (SCORE2) as a risk model that estimates the 10‐year risk of CVD in adults without previous CVD or diabetes^44^ and a model incorporating MASLD was conducted. For quantifying the improvement in predictive accuracy, the change in Harrell's C‐index and the net reclassification index (NRI) were calculated to assess the extent to which MASLD enhances patient risk classification (*Table* *4*). FLI has independent predictive value after adjusting for SCORE2 and relevantly improves the C‐index. The NRI indicated a correct reclassification by including FLI by approximately 20% for both MACE and eMACE.

**Table 4:** Metabolic dysfunction‐associated steatotic liver disease in cardiovascular risk stratification

	FLI	SCORE2 algorithm	SCORE2 algorithm + fatty liver index	Δ C‐index *p*‐value^a^
MACE				
HR (95% CI)	1.017 (1.013–1.020), *p* < 0.0001	1.006 (1.004–1.008), *p* < 0.0001	1.016 (1.012–1.019), *p* < 0.0001	
C‐index	0.644	0.607	0.646	0.0016
NRI (95% CI)			0.22 (0.17–0.27), *p* < 0.0001	
eMACE				
HR (95% CI)	1.015 (1.012–1.018), *p* < 0.0001	1.004 (1.002–1.006), *p* < 0.0001	1.014 (1.011–1.017), *p* < 0.0001	
C‐index	0.63	0.58	0.627	**<0.0001**
NRI (95% CI)		–	0.19 (0.138–0.23), *p* < 0.0001	

CI, confidence interval; eMACE, extended major adverse cardiovascular events; HR, hazard ratio; MACE, major adverse cardiovascular events; NRI, net reclassification index; SCORE2, Systematic COronary Risk Evaluation 2.

^a^ Δ C‐index *p*‐value between SOCRE2 algorithm and SCORE2 algorithm + fatty liver index.

## Discussion

In this cohort study of 15 010 participants in the GHS, hepatic steatosis was highly prevalent and the FLI was positive in 37%. The FLI is a validated and well‐established non‐invasive test with an accuracy of 0.84.^35^, ^45^, ^46^ The estimated prevalence of 37% is in line with previously published data on the prevalence of MASLD in Europe and Germany.^45^, ^47^ Accordingly, data from the past decades have also shown a marked increase in the prevalence of NAFLD from 15% in 2005 to 25% in 2010.^48^ In a population‐based study in young adults in the UK, the prevalence of steatosis was 20.7%, measured by transient elastography.^49^ Data from the National Health and Nutrition Examination Survey (NHANES) database in the United States found a prevalence of 40.4% of hepatic steatosis in young adults utilizing transient elastography.^50^ In the MASLD subgroup, men and elderly people were overrepresented and comorbidities including arterial hypertension and diabetes were found more frequently. These characteristics represent well‐known risk factors for the development of MASLD, and the current findings underline the strong association of MASLD and metabolic comorbidities.^1^, ^2^, ^11^ Comparable epidemiologic data on liver fibrosis are scarce, but the prevalence of MASH in Western Europe is estimated to be about 4%.^1^

In this cohort study the presence of advanced fibrosis as determined by the FIB‐4 score with a cut‐off >2.67 was 0.8%. This test has been suggested as enrichment tool for referral pathways^51^ but will miss patients with early stages of hepatic fibrosis (F1–F2).^52^

In the current analysis, MASLD patients were significantly more often affected by MACE and exhibited a more than two times higher all‐cause mortality rate. After adjustment for age and gender, MASLD remained an independent risk factor for incident cardiovascular events on regression analysis. With the analysis, we were able to show that there is a connection between hepatic steatosis and MACE and eMACE, with various factors influencing this, such as hypertension, dyslipidaemia and diabetes mellitus. Hepatic steatosis, if present without these factors, represents a higher risk factor for MACE, which suggests that there might be different aetiologies included in the sample leading to the changes in the liver that are associated with different risks for MACE. The FLI score, and thus the MASLD diagnosis, therefore appears to group together different subgroups of diseases.

In the current analysis, MASLD confers a risk for developing MACE and eMACE with an approximately 1.02 times larger effect in obese (BMI ≥30 kg/m^2^) than in non‐obese individuals. This is a central observation and could be related to a number of factors. The concept of metabolic inflammation^53^ has been identified as a driver of the disease and outcomes and its impact is likely stronger in obese versus non‐obese participants. Additionally, the non‐obese group does include patients with a BMI defining normal weight (≤25 kg/m^2^). Within this group, that was formerly known as lean NASH, the degree of heterogeneity is larger. Still, based on its simplicity and availability, FLI can serve as a risk stratification tool in the general population to be used in clinical practice.

Our findings on the negative effect of hepatic steatosis on CVD complement a series of studies in several countries with comparable results.^30^, ^54^ In the United States, Meyersohn *et al*.^55^ showed in a smaller cohort study of 3756 participants, that hepatic steatosis detected by computed tomography imaging was associated with MACE independently of other cardiovascular risk factors. In South Korea, Moon *et al*.^46^ analysed data of 351 068 citizens from a nationwide health screening database and found that individuals with MASLD (FLI >60) were at an increased risk of developing CVD. In Sweden, Simon *et al*.^56^ recorded MACEs in a population‐based cohort of 10 422 adults with histologically confirmed MASLD and observed that the rate of incident MACE increases with MASLD disease stage. Data from Germany are scarce. In a smaller cohort of 1096 adults from the Study of Health in Pomerania in Northeastern Germany, data analysis showed an association between MASLD and cardiac remodelling detected by magnetic resonance imaging.^47^

To assess the clinical utility of MASLD in cardiovascular risk stratification, we evaluated whether adding MASLD to established risk prediction models improves predictive performance. The models show an improved risk classification when adding FLI to the SCORE2. FLI has independent predictive value after adjusting for SCORE2 and relevantly improves the C‐index. The NRI indicated a correct reclassification by including FLI by approximately 20% for both MACE and eMACE. The models show that FLI retains its predictive power despite the SCORE2 score. Therefore, FLI offers additional prognostic value beyond traditional risk factors and justify its inclusion in cardiovascular risk assessment.

In contrast, the preexistence of liver fibrosis did not confer an increased risk of MACE, but contributed to an increased risk of all‐cause mortality. This might be due to the fact that the presence of fibrosis indicates more advanced disease stages, in which morbidity and mortality are mainly caused by the liver disease itself and its various complications including portal hypertension, decompensated cirrhosis ad hepatocellular carcinoma.^5^, ^27^, ^57^, ^58^, ^59^, ^60^ In fact, advanced liver fibrosis is known to be a strong predictor of mortality in patients with MASLD.^61^, ^62^

This study has limitations as it was based on a regional sample from the Mainz‐Bingen area in Rhineland‐Palatinate, Germany and may not be directly representative for other countries or other regions in Germany. When assessing the external validity or generalizability of the study, it must also be considered that persons with severe health restrictions and persons without sufficient knowledge of German were fundamentally excluded from participation in the study. Furthermore, a history of liver disease was self‐reported at baseline and in the current study, the reported prevalence of 8% of underlying liver disease is likely an overestimation. The presence of MASLD was defined as the presence of steatosis of the liver with a positive FLI and the exclusion of other chronic liver disease or high alcohol consumption. We did not utilize imaging techniques like sonography, transient elastography or magnetic resonance imaging as reference standard to identify steatosis. Also, alcohol consumption was self‐reported and thus underreporting could have occurred. We choose waist‐to‐height ratio rather than BMI to overcome limitations in the categorization of obesity and body fat distribution.

Our findings support the concept that MASLD is a multisystem disease with a complex pathophysiology affecting not only the liver but also the cardiovascular system.^63^ The effect of MASLD on incident CVD could in part be mediated through atherogenic dyslipidaemia as well as a systemic low‐grade inflammation, which is seen in most MASLD patients.^64^, ^65^ This contributes to chronic vascular inflammation, endothelial dysfunction and the remodelling of blood vessels with formation of atherosclerotic plaques.^66^

The results of this large, prospective, population‐based study underline the high burden of disease related to MASLD in the German general population and highlight the need for targeted therapies as well as efficient primary and secondary prevention strategies. International expert groups of cardiologists and hepatologists ask for more awareness among physicians and a standardized evaluation of cardiovascular risk in MASLD patients.^67^, ^68^, ^69^ The growing understanding of MASLD pathophysiology has led to new therapeutic approaches, which are aimed at ameliorating the metabolic function and suppress chronic inflammation. Existing drugs, which might have a beneficial effect on both MASLD and CVD, include for example incretins or the thyroid hormone receptor beta‐agonist resmetirom.^70^, ^71^

With the emergence of these therapies, screening tools to identify individuals with advanced hepatic fibrosis related to MASLD are implemented. As these patients are increasingly identified, our data support the concept of screening for CVD to prevent incident MACE. In this context, the FLI that was used in this cohort study represents an easy, inexpensive, broadly available and non‐invasive diagnostic tool, which is based on laboratory measurements (triglycerides, gGT) and basic physical examination (BMI, WC). Interestingly, Zou *et al*.^45^ showed in a UK Biobank analysis that the FLI not only predicts MASLD but is also an independent risk factor for CVD. To replace FLI and FIB‐4 liver elastography is available today to diagnose MASLD with high accuracy.^37^ Nonetheless, the FLI has been included in regional guidelines and is a helpful diagnostic tool at a low resource level to identify patients with MASLD.

In conclusion, MASLD was shown to be an independent risk factor for MACE and MASLD patients displayed a significantly increased risk of all‐cause mortality in the general population in Germany. Further investigations and longitudinal data over a more extended period are needed to clarify the role of MASLD on cardiovascular health.

## Supporting information


**Appendix S1.** Supporting Information.
