# Supplementary material for: Incidence of major cardiovascular events in patients with metabolic dysfunction‐associated steatotic liver disease in the general population
Source: Eur J Heart Fail. 2025 Sep 26;27(11):2490–500. doi: 10.1002/ejhf.70053 (PMC12765464; doi:10.1002/ejhf.70053)
Supplement: Supplementary file 1 — Appendix S1. Supporting Information. [file EJHF-27-2490-s001.docx]

Supplement Table S1. Baseline characterization of study cohort standardized according to European Standard Population 1976

| **Parameter** | **All** | **Men** | **Women** | **p** |
| --- | --- | --- | --- | --- |
| Age [y] | 51.7 (±10.7) | 51.7 (±10.7) | 51.7 (±10.7) | 0.75 |
| Female sex | 7371 (50.5) |  |  |  |
| BMI [kg/m^2^] | 26.6 (23.6; 29.8) | 27.1 (24.7; 30.0) | 25.4 (22.5; 29.4) | <0.0001 |
| Waist circumference [cm] | 93.5 (± 13.9) | 98.6 (± 12.1) | 88.5 (± 13.7) | <0.0001 |
| Obesity | 3488 (23.9) | 1824 (25.2) | 1665 (22.6) | 0.0002 |
| Diabetes mellitus type 2 | 1080 (7.4) | 650 (9.0) | 430 (5.9) | <0.0001 |
| Hypertension | 6360 (43.6) | 3498 (48.4) | 2861 (38.9) | <0.0001 |
| Dyslipidaemia | 4574 (31.4) | 2932 (40.7) | 1642 (22.3) | <0.0001 |
| Metabolic syndrome | 2986 (20.5) | 1911 (26.4) | 1075 (14.6) | <0.0001 |
| Chronic liver disease | 1094 (7.6) | 507 (7.1) | 587 (8.0) | 0.029 |
| Cancer | 1095 (7.5) | 449 (6.2) | 646 (8.8) | <0.0001 |
| Hyperuricemia | 1148 (8.1) | 784 (11.2) | 363 (5.1) | <0.0001 |
| Smoker | 3063 (21.0) | 1631 (22.6) | 1432 (19.5) | <0.0001 |

Values are presented as mean (+/- SD), median (interquartile range), or counts (%), as appropriate.

^*^ high alcohol consumption: defined as an daily intake of ≥ 40 g for women and ≥ 60 g for men
